# Monitoring Sodium Content in Processed Foods in Argentina 2017–2018: Compliance with National Legislation and Regional Targets

**DOI:** 10.3390/nu11071474

**Published:** 2019-06-28

**Authors:** Lorena Allemandi, Maria Victoria Tiscornia, Leila Guarnieri, Luciana Castronuovo, Enrique Martins

**Affiliations:** Fundacion Interamericana del Corazon Argentina, Arévalo 2364, C1425 Buenos Aires, Argentina

**Keywords:** sodium reduction, processed foods, public health policies, Argentina, Latin America

## Abstract

Sodium intake in Argentina has been estimated to be at least double the dose of 2000 mg/day recommended by WHO, mostly coming from processed foods. Argentina is one of the few countries in the world that have regulated sodium content in certain food products. This study presents an assessment of sodium content in a selection of food groups and categories as reported in the nutrient information panels. We surveyed 3674 food products, and the sodium content of 864 and 1375 of them was compared to the maximum levels according to the Argentinean law and the regional targets, respectively. All food categories presented high variability of sodium content. Over 90% of the products included in the national sodium reduction law were found to be compliant. Food groups with high median sodium, such as condiments, sauces and spreads, and fish and fish products, are not included in the national law. In turn, comparisons with the lower regional targets indicated that almost 50% of the products analyzed had sodium contents above the recommended values. This evidence suggests that enhancing sodium reduction in processed foods may be a necessity for public health objectives and it is also technically feasible in Argentina.

## 1. Introduction

Excess sodium intake has been linked to raised blood pressure and cardiovascular diseases (CVD) [1,2,3,4], directly causing 5.6% of premature deaths and 2.4% of disability worldwide [1,5,6]. CVD is the leading cause of death in the general population of Argentina, responsible for 27% of deaths per year [7], of which over one-third are attributable to hypertension [8]. Moreover, objective measurements conducted as part of the 2018 National Risk Factor Survey indicate that hypertension affects 40.6% of the adult population in the country [9]. Similar to what has been reported for other countries in the region [10], sodium intake in Argentina has been estimated to be at least double the dose of 2000 mg/day recommended by the World Health Organization (WHO), of which 50–70% comes from processed foods [11,12].

Sodium reduction policies are cost-effective in reducing the global burden of CVD [13,14], as has been proven in countries such as Finland, France, and the UK, where sodium reduction plans have resulted in reductions of dietary sodium intake [15]. Research conducted in Argentina has already established the feasibility of sodium reduction in processed foods in the country (10). A reduction of 3 g of salt in the Argentinean diet has been estimated to decrease the prevalence of CVD by over 20%, also contributing to reduce mortality rates from heart disease by 19.9% [16].

Argentina is one of the few countries in the world where the government has taken direct action to tackle this public health issue. In December 2013, Argentina passed Act 26,905, which sets the maximum sodium levels in three processed food groups—meat products, farinaceous foods, and soups, bouillons, and dressings—and implied reductions of sodium content by 5% to 18% over the following two years [17]. The law entered into force in December 2014, a year after its enactment, allowing food companies up to 12 months to achieve full compliance. Furthermore, in the Latin American and Caribbean (LAC) region, regional targets for sodium content were set for 11 food categories by the Salt Smart Consortium, a group of government and non-governmental organizations and food companies. LAC countries were expected to have met these targets by December 2016 [18].

In 2014, we conducted a study to compare the baseline sodium levels of processed foods in Argentina as reported in food labels with the maximum levels set by the national law [19]. The objectives of this paper were: a) to assess the current sodium levels in a variety of processed food groups and categories available in the Argentinean market; b) to monitor compliance with the maximum levels set forth by Act 26,905, four years after its entry into force; and c) to compare the current sodium content levels with the regional targets. In this study, we measured progress in implementing the sodium policy in Argentina and we analyzed sodium levels in consideration of the regional recommendations for salt/sodium values.

## 2. Materials and Methods

### 2.1. Study Design

This was a cross-sectional, systematic survey to compare the sodium content of processed foods in Argentina as reported in nutrition information panels with the maximum values set in Act 26,905 in 2013 and in the regional targets. This study was part of the research project “Scaling Up and Evaluating Salt Reduction Policies and Programs in Latin American Countries”, a collaborative regional study that includes Argentina, Brazil, Peru, Paraguay, and Costa Rica.

### 2.2. Data Collection

Data were collected between August 2017 and May 2018 in four stores corresponding to three leading retailers in Argentina, as informed by market share reports; together, these companies account for over 12% of grocery retail market [20]. Three stores were located in the province of Buenos Aires and one in Buenos Aires city. Data collection was conducted by members of the research team on-site, with written store management approval. Each product was surveyed using the Food Label Information Program (FLIP, Huntington Beach, CA, USA) developed by the University of Toronto [21]. FLIP is a food composition database software (web and mobile) that provides a shorter and more efficient food collection and data processing approach. Data collection consisted of scanning the bar code of each product and taking photographs of all sides. This information was then uploaded to the FLIP database. For each product, the manufacturer, brand, and product names, serving size, container size, and complete nutritional information of the product as consumed (serving/g or mL) were entered. Food groups and categories included in the analysis were defined prior to data collection (see Section 2.3). Data collectors walked along and observed all the aisles and product displays in each store to ensure that all products available for purchase and belonging to the food categories of interest were recorded, resulting in a total sample of 3674 products. Food products sold at multiple retailers were captured only once. When multiple sizes of a product were available, only one size was sampled, but all flavors and varieties of each product were surveyed.

### 2.3. Definition of Food Groups and Categories

**Figure 1:**
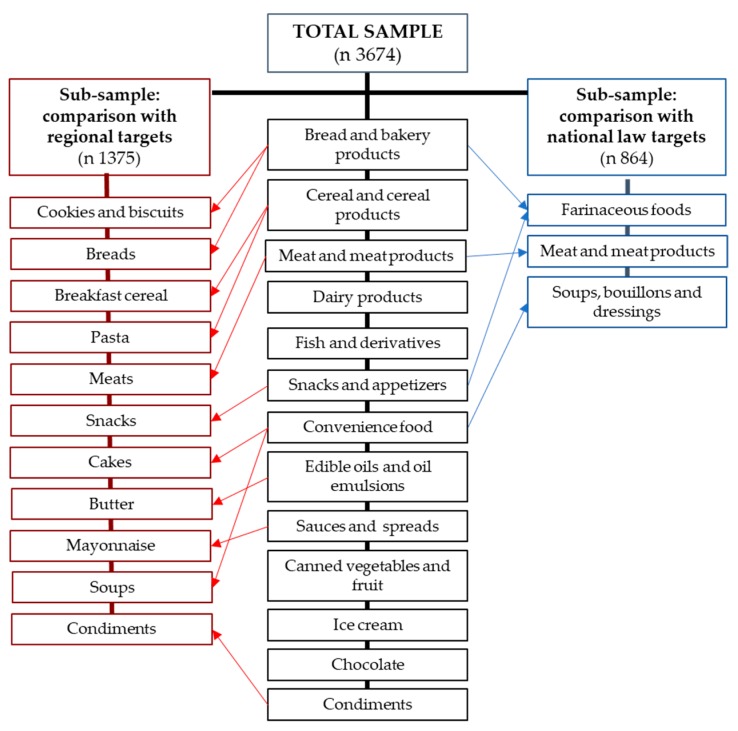
Food groups included in the total sample and their relationships with food groups whose maximum sodium levels are set by national law and regional targets.

Food groups were defined to include products manufactured from the same raw material and with similar nutritional content (e.g., bread and bakery products, dairy products, etc.). Food categories included products with the same manufacturing process (e.g., biscuits, bread, etc., within the bread and bakery products groups) [22]. The final food categorization system included 14 food groups: bread and bakery products, cereals and cereal products, convenience foods, dairy products, meat and meat products, fish and derivatives, snacks and appetizers, edible oil and oil emulsions, sauces and spreads, beverages, canned vegetables and fruit, ice cream, chocolate and seasonings (Figure 1). These groups were also classified into 40 categories.

For the comparison of the surveyed products against the sodium content targets established by Act 26,905 in 2013, we matched food groups and categories from our database with the food groups and categories included in the law: meat and meat products, farinaceous foods, and soups, bouillons, and dressings (Figure 1). These groups include 27 categories: cooked, dry and fresh sausages, luncheon meat, hamburgers, breaded chicken products, bran crackers, non-bran crackers, snack crackers, corn flour snacks, cheese puffs, cheese-flavored sticks, potato chips, unsalted potato chips, salted peanuts, nachos, other snacks, dry sweet biscuits, filled sweet biscuits, wholemeal bread, white bread, hot dog buns, hamburger buns, bouillon cubes and granulated soup, clear soup, cream soup, and instant soup.

For the comparison against regional targets, we considered the products belonging to the 11 categories (Figure 1) and 17 sub-categories defined in the Salt Smart Consortium Consensus of 2015 [18]. These categories are dry soups and noodles in broth as consumed, meats and cooked, raw, or processed sausages, cured dry meats and meats preserved at room temperature, breaded meat and poultry, bread, mayonnaise, sweet cookies and cookies, flavored cookies and crackers, mixes for aerated cakes, breakfast cereals, butter, snacks, shelf-stable dry or uncooked pasta and noodles, pasta and noodles as consumed, meat and fish seasoning, and bouillon cubes and powders. This analysis was performed against two different targets. The “regional target” for salt/sodium concentration is the highest allowed sodium content per 100 g of food in any of the countries that have set legal limits in the Americas. In turn, the “lower target” is the lowest value that exists in this same range. The regional target thus implies a moderate restriction, while the lower target is consistent with a more stringent approach towards sodium reduction in the region.

### 2.4. Data Analysis

The sodium content in foods was obtained from the nutrition facts table (mg/serving) and was converted to standardized units (mg/100 g) considering the products as consumed. Median values and 25 and 75 percentiles were used to characterize the distribution of the data set in each food group and category. The mean and the range are included as a reference; the percentage coefficient of variation (C.V.%) is provided as an alternative index of dispersion.

The median sodium content of products belonging to food groups/categories considered in the national law was compared with the sodium targets established by the law in 2013 and the regional and lower targets. We present the percentage of products in each category that exceed the maximum thresholds in each system. Finally, we show the percentage of products that meet the legal target and of those that are above the target by <25%, between 25% and 50%, and by >100%. All data analyses were conducted using SPSS v.20 software.

## 3. Results

### 3.1. Sodium Content by Food Group and Category

Most of the total sample (n = 3674) consisted of bread and bakery products (16%), dairy (16%), non-alcoholic beverages (11%), cereal and cereal products (11%), and convenience foods (10%) (% were calculated as the number of products in each category/total number of samples) (Table 1). The five groups with the highest sodium content were condiments (median: 16,690 mg/100 g), sauces and spreads (median: 866.7 mg/100 g), meat and meat products (median: 843.8 mg/100 g), snacks (median: 683.7 mg/100 g), and fish and fish products (median: 411.7 mg/100 g). Chocolate (median: 100.7 mg/100 g), ice cream (median: 58.5 mg/100 g), and non-alcoholic beverages (median: 17.0/100 g) had the lowest median sodium content.

Within food groups, the categories with the highest median sodium content were bouillon cubes and powders (median: 20386.7 mg/100 g), meat and fish seasoning (median: 13250.0 mg/100 g), appetizers (median: 1600.0 mg/100 g), sausages and luncheon meat (median: 992.5 mg/100 g), and dressings (median: 950 mg/100 g) (Table 1). Sodium content was very variable among products belonging to the same category. Maximum variability was reported for other fish products (range: 0.0–7386.67 mg/100 g, C.V.: 213.7%), canned vegetables (range: 0–4760 mg/100 g., C.V.: 181.5%), butter (range: 0–920 mg/100 g, C.V.: 135.7%), and frozen vegetables (range: 0–575 mg/100 g, C.V.: 130%) (Table 1).

### 3.2. Comparison of Sodium Content Against the Maximum Levels Set by National Act 26,905

**Figure 2:**
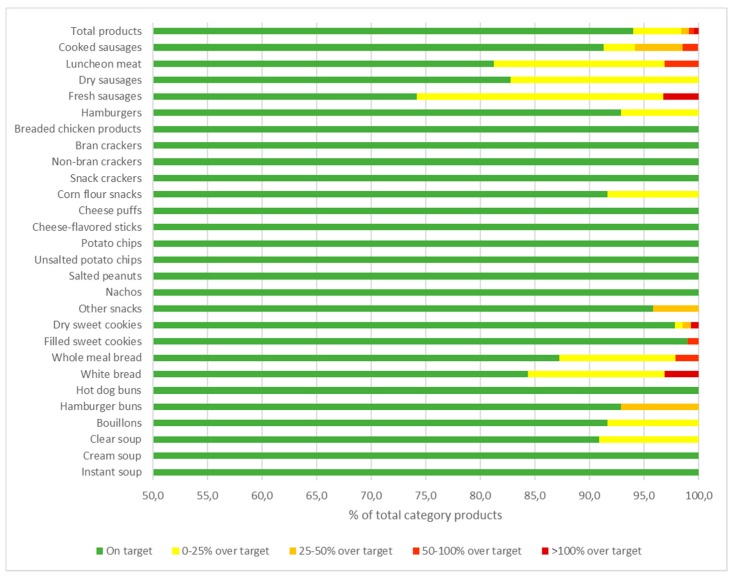
Food product categories compared with the National Act 26,905 maximum sodium levels. In green, % of products with a sodium content below the allowed maximum sodium level. In yellow, orange, red, and dark red, % of products with a sodium content above the allowed sodium level by 0–25%, 25–50%, 50–100%, or more (n = 864).

There were 864 products in our database (n = 3674) that were included in the food groups and categories regulated by Act 26,905. Most of them belong to the farinaceous food group (n = 577), followed by meat and meat products (n = 220) and soups, bouillons, and dressings (n = 67). Although the observed median values were below the sodium targets in all product categories, 14 of the 27 categories considered in this analysis included one or more specific products whose sodium levels were above the category target. Of the 864 products analyzed, 5.7% (n = 49) were found to exceed the sodium content targets set by law (Table 2 and Figure 2).

Among meat and meat products, 12.7% of the products were above the allowed maximum, as were 3.1% of farinaceous products and 4.5% of soups, bouillons, and dressings. Within meat and meat products, only processed poultry was found to be in full compliance with the law, i.e., all products were below the legal threshold. The category of uncooked sausages comprised most of the non-compliant products (25.8%), followed by luncheon meat (18.8%) and dry sausages (17.2%). Among the farinaceous group, the categories with most of the non-compliant products were white bread (15.6%), wholemeal bread (12.8%), and corn flour snacks (8.3%). Finally, only three soups, bouillons, and dressing products were found to be above the established targets; two of them were bouillon (8.3%), and one was clear soup (9.1%) (Table 2 and Figure 2). Most products with sodium contents above the legal target exceeded it by less than 25% (n = 38); six and four products were above the legal target by 25 to 50% and 50 to 100%, respectively. These included fresh and cooked sausages, luncheon meats, and dry sweet cookies. Only three product categories (white bread, fresh sausages, and dry sweet cookies) presented products (one per category) with sodium contents that exceeded the legal limit by 100% or more (Figure 2, Table S1).

### 3.3. Comparison of Current Sodium Levels in Argentina with Regional Sodium Targets

This analysis was performed on 1375 of the total 3674 surveyed products from 11 food groups and 17 categories included in the regional targets. Of these products, 12.7% were found to exceed the regional target for sodium content, including condiments (29.3%), cookies and biscuits (19.8%), pasta and noodles (12.9%), soups (11.5%), and meat products (9.5%), among others. Bouillon cubes and powders (50%), dry cured meats and meats preserved at room temperature (25%), cookies and crackers (21.2%), dry/uncooked pasta and noodles (13.1%), meats and raw, cooked, and processed meats and sausages (6.9%) were among the categories with the highest number of products exceeding the regional sodium targets (Table 3).

When compared to the lower targets, 49.6% of the total sample had sodium contents above the recommended maximum values. The 11 food groups had at least a few products with above-target sodium levels. The categories in which most of the products were off target were mayonnaise (97.1%, n = 34), mixes for aerated cakes (90.9%, n = 10), noodles in broth (88.9%, n = 8), meats and raw, cooked, and processed sausages (80.5%, n = 140), dry cured meats and meats preserved at room temperature (75%, n = 30), snacks (69.1%, n = 96), bouillon cubes and powders (68.2%, n = 15), flavored cookies and crackers (60%, n = 15), cookies and sweet cookies (53.4%, n = 194), and breaded meat and chicken products (52.9%, n = 9) (Table 3).

## 4. Discussion

This study is the second analysis of sodium content in processed foods as reported in the nutrition information panels performed in Argentina. It is part of an ongoing effort to independently monitor the sodium content of processed foods over time and the implementation of the national law.

Our results indicate that condiments, sauces and spreads, meat and meat derivates, snacks and appetizers, and fish and fish products have the highest sodium contents among all food groups included in the study. The high variability of sodium content found within each category also shows the technical feasibility of reducing sodium levels within these food groups, as has been found in other studies around the world [23,24].

Although Argentina is one of the pioneering countries in the LAC region in promoting mandatory policies to reduce sodium intake, there are still products that fail to meet the maximum levels for sodium content established by the law, over four years after its entry into force. Approximately 6% of the products studied were found to exceed the maximum sodium contents allowed, mostly meat and meat products and breads, which constitute the most important sources of sodium intake from processed foods in the country [12].

The results from the baseline study published in 2015 [19] are not strictly comparable to this research due to differences in methodology, mainly, a much smaller sample size and the use of different criteria to group the food products into categories. However, they suggest that there has been little change in median sodium contents in the products included in the law. About 85% of the surveyed products where already on target in February 2014, before the law entered into force. This is probably due to the fact that the maximum sodium levels set by law were the same as those established in previous voluntary agreements with the food industry [19]. Even if full compliance with the maximum levels were achieved, the effective sodium reduction would reach a maximum of about 30 mg/day [12], which would not have a considerable impact on the overall sodium intake.

There are few countries around the world that have established legal maxima to sodium content in processed foods, and in most cases, these regulations apply to specific food categories, i.e., bread [25,26]. To date, South African legislation is the most similar to the Argentinean policy, including a comparable range of food products (bread, snacks, processed meat products, soups and dressings). While over 90% of the regulated products were found to have sodium levels below the allowed maxima in Argentina, only 67% of the analyzed products in South Africa were compliant with their national legislation [27]. However, the South African approach to sodium regulation has been more stringent, since the maximum sodium contents allowed for several critical food categories—e.g., bread, processed meats— are much lower than those set by the Argentinean law analyzed in this study. In fact, they are very close to the lower regional targets established by Pan American Health Organization (PAHO). For example, the maximum sodium content allowed for bread in South Africa as of June 2019 is 380 mg/100 g, and that for different categories of processed meat products is between 600 and 850 mg/100 g [27].

Despite the shortcomings described above, the government does have full authority to revise the reduction targets and broaden the law’s scope to include other food categories. Some steps have been taken to further reduce the restrictions already in place for sodium content. In September 2018, a new regulation updated the sodium reduction targets for meat and farinaceous products, lowering them by approximately 5%; these targets will enter into force in March 2020, since food companies have up to 18 months to meet these new maximum levels [28]. Similarly, targets for bouillons and soups were lowered by 5% to 6% in the new regulation passed in February 2019. This legislation also incorporates ketchup and mayonnaise in the list of products whose sodium content is regulated by law, setting a maximum level of 833 and 980 mg of sodium per 100 g of product [29]. While the incorporation of these new products is positive from a public health perspective, categories such as frozen breads (hamburger and hot dog buns) have been excluded from these updates. Although progressively lowering the maximum levels is surely necessary, further reduction efforts must also be directed at setting legal targets for other food groups and categories. For instance, food groups such as condiments, sauces and spreads, and fish and fish products, which have high median sodium levels, are currently not included in Act 26905. To ensure substantial sodium intake reductions, it is essential to incorporate targets for these and other food groups and categories, such as cheese and puff pastry, that have been proven to contribute significantly to sodium intake in the country [12].

When compared to the regional targets, 87.3% of the products analyzed were found to be on target. This is not surprising, considering that Argentina’s national targets were a significant input to develop the regional recommendations, and most products measured in Argentina are already compliant. However, when compared to the more stringent regional targets (“lower targets”), almost half of the products were above the recommended maximum for their category. This situation will not be reverted when the new national maximum levels are fully achieved. For example, the lower target for breads and snacks are 400 mg and 530 mg/100 g, respectively [18], while the updated maximum levels allowed in Argentina since September 2018 are 476 mg and 900 mg/100 g [26]. The maximum sodium level set in February 2019 for mayonnaise is well below the regional target (1050 mg/100 g) [28] but above the lower target (670 mg/100 g). A similar case can be made for soup, whose updated maximum sodium levels is 330 mg/100 mL of product, which falls in between the regional (360 mg/mL) and lower (306 mg/mL) targets. This suggests that, by the time both new regulations are fully in force (March–August 2020), the lower sodium targets will still not have been met by almost half of all products included in the affected categories. Moreover, the results obtained when comparing the sodium content in Argentinean products with the regional sodium targets are very similar to those obtained by a regional study, in which 82% and 47% of food products from 14 LAC countries (including Argentina) were found to meet the regional and lower targets, respectively [30].

This study does present certain limitations, mainly, the fact that it is not representative of all products available in the Argentinean market, such as those sold in informal markets and bakery products; also, inter-store or regional variations in product availability were not addressed because of the small number and the location of the stores surveyed—Buenos Aires city and its suburbs. While the stores selected belong to three of the largest food retailers, there are no retailers with country-wide presence in Argentina [20]. Similarly, this study did not considered market share data for each of the different products analyzed; therefore, we cannot ensure that the sample is representative of the majority of products purchased in each food category. Another limitation is that the sodium content that was considered for the analyses relied on food labelling, without chemical analysis verification. The food Argentinean Food Code allows a 20% difference between food label and laboratory values.

However, this analysis represents a valuable tool to independently monitor the policy currently in force and identify weaknesses and opportunities for improvement. The lessons learned from this monitoring process can also facilitate similar sodium reduction initiatives in other countries in the Americas. However, to properly evaluate the effectiveness of this policy, specific studies must be conducted to assess the policy’s real impact on sodium intake in the population, as well as to identify reductions (or increases) in the burden of CVD attributable to sodium consumption. Finally, for these food reformulation efforts to succeed in effectively reducing sodium intake, it is essential to develop other policies that continue to increase awareness of these and other healthy nutrition issues among the population. Such measures include, but are not limited to, the implementation of straight-forward front-of-pack labelling, the restriction of the marketing of unhealthy foods, especially those directed at children, and the development of policies to increase the availability and affordability of fruits and vegetables.

## Figures and Tables

**Table 1:** Sodium content of processed foods in Argentina by food group and category (n = 3674).

Food Group	Food Category	Products (n)	Sodium Content (mg/100 g)					
			Mean	Median	Range (Min–Max)	%ile 25	%ile 75	C.V. (%) *
Bread and bakery products	Bread	110	442.7	443.0	1.08–1030	395.0	500.0	28.4
	Toast	23	439.5	500.0	15.2–783.33	66.7	783.3	75.6
	Biscuits	363	311.0	278.6	0–1120	160.0	430.0	68.0
	Bakery products	101	299.5	250.0	15–960	170.8	392.5	68.7
**Total bread and bakery products**		**597**	**338.3**	**316.7**	**0–1120**	**184.4**	**478.0**	**62.1**
Cereal and cereal products	Cereal bars	40	160.3	159.0	0–604.35	105.3	193.5	62.7
	Breakfast cereal	85	282.7	250.0	0–810	126.7	416.7	69.1
	Pasta and noodles	259	258.7	36.0	0–1075	10.0	537.0	113.9
	Soy-based products	24	411.2	424.0	18.82–832.94	381.9	459.3	47.3
**Total cereal and cereal products**		**408**	**263.0**	**181.2**	**0–1075**	**12.5**	**487.1**	**99.8**
Convenience foods	Puff pastry for pies	27	712.8	673.3	50–1200	620.0	850.0	30.3
	Puff pastry for empanadas	38	732.1	693.1	450-1200	620.6	836.5	25.7
	Soup	52	300.3	292.0	40–625	228.1	306.3	37.2
	Bouillon cubes	24	339.0	378.4	32–450	284.0	407.6	32.2
	Pizza	21	709.1	610.7	178.33–1332.17	562.2	843.9	43.3
	Ready-made meals	16	206.6	210.8	18.16–474.39	32.8	338.3	74.4
	Pre-cooked meals	48	444.9	442.2	51.76–1754.67	282.4	523.6	73.2
	Pre-mixtures	25	421.5	406.3	17.65–1037.14	260.1	520.0	55.2
	Instant dessert mixtures	80	77.1	72.5	11.72–176	43.3	107.5	52.2
	Frozen vegetables	39	79.3	60.0	0–575	20.0	86.0	130.0
**Total convenience foods**		**370**	**351.8**	**293.2**	**0–1754.7**	**86.0**	**536.3**	**86.4**
Dairy	Cheese	316	628.5	583.3	13.33–2190	386.7	815.0	57.4
	Dairy-based desserts	69	84.6	93.6	35–110.53	80.4	96.3	26.5
	Yoghurt	142	54.5	50.0	0–138.89	43.0	61.7	37.2
**Total dairy**		**527**	**402.6**	**300.0**	**0–2190**	**64.0**	**630.0**	**97.5**
Edible oils and oil emulsions	Butter	19	145.2	92.0	0–920	92.0	140.0	135.7
	Margarine	19	442.1	540.0	0–810	200.0	650.0	52.1
**Total edible oils and oil emulsions**		**38**	**293.7**	**200.0**	**0–920**	**92.0**	**547.5**	**88.4**
Fish and derivatives	Canned tuna	32	389.0	360.8	221.67–735	275.0	467.9	34.3
	Canned mackerel	8	217.3	218.3	98.33–343.33	175.0	255.0	34.0
	Other fish **	64	900.0	427.5	0–7386.67	228.8	685.0	213.7
	Canned sardines	4	393.8	412.5	173.33–576.67	198.8	570.0	50.9
	Breaded fish products	15	487.4	496.2	149.23–887.69	293.8	629.0	43.3
**Total fish and derivatives**		**123**	**655.9**	**411.7**	**0–7386.7**	**248.3**	**588.3**	**188.4**
Meat and meat products	Hamburger	42	771.0	800.0	395–967.27	750.0	843.8	12.9
	Luncheon meat and sausages	172	1079.3	992.5	115–3622.5	785.0	1281.9	47.9
	Spreads	15	760.0	750.0	260–1050	700.0	950.0	26.0
	Breaded chicken products	17	468.7	516.9	46.15–617.69	398.5	600.0	35.0
	Others	7	660.6	702.3	398.46–843.31	476.9	843.3	26.2
**Total meat and meat products**		**253**	**956.5**	**843.8**	**46.2–3622.5**	**702.4**	**1092.5**	**175.1**
Snacks and appetizers	Snacks	164	634.2	608.0	0–1236.67	500.0	758.0	36.5
	Appetizers	76	1590.7	1600.0	2–2885	1164.0	2258.8	47.2
**Total snacks and appetizers**		**240**	**937.1**	**683.7**	**0–2885**	**552.0**	**1216.0**	**68.4**
Sauces and spreads	Sauces	46	348.4	335.0	0–1320	213.8	400.0	67.9
	Dressings	168	1493.4	950.0	0–7333.33	783.3	1558.7	101.3
**Total sauces and spreads**		**214**	**1247.3**	**850.0**	**0–7333.3**	**405.8**	**1304.2**	**114.2**
Beverages	Non-alcoholic beverage	419	19.4	17.0	0–77	9.0	27.0	73.8
Canned fruit and vegetables	Canned vegetables	147	251.9	196.9	0–4760	63.3	261.5	181.5
	Canned fruit	36	10.5	5.4	0–40	0.0	20.0	108.2
**Total canned fruit and vegetables**		**183**	**204.4**	**160.8**	**0–4760**	**19.3**	**233.1**	**205.8**
Chocolates	Alfajores	40	109.8	106.4	0–320	64.1	142.0	61.6
	Chocolate icing	10	68.2	54.0	23.6–132	34.8	108.5	59.8
	Dipping chocolate	1	276.0	−	−	−	−	−
	Chocolate bars	59	106.7	112.0	0–280	94.5	136.0	47.5
	Cocoa powder	15	56.9	63.5	0–73.5	54.5	70.0	40.8
	Others	13	90.4	125.0	0–143.3	38.8	138.5	63.0
**Total chocolates**		**138**	**99.1**	**100.7**	**0–320**	**63.5**	**132.0**	**59.0**
Ice cream	Ice cream	123	52.9	58.5	0–190.8	31.7	68.3	60.8
Condiments	Seasonings	19	14,095.2	13,250.0	7828.57–23,493.33	10,145.8	17,100.0	31.5
	Bouillon cubes and powders ***	22	20,308.9	20,386.7	2000–33,813.3	13,378.9	25,953.3	39.2
**Total condiments**		**41**	**17,429.4**	**16,960.0**	**828.5–33,813.3**	**12,325.3**	**22,600.0**	**41.4**

* Coefficient of variation; ** Examples of products included in the “other fish” category are frozen surimi, ready-made shellfish stew, and canned salmon in oil. *** Bouillon cubes within the condiments group are added directly to food preparations and are not reconstituted with water, like bouillon cubes within the convenience food groups.

**Table 2:** Comparison of the observed sodium content with the maximum levels set forth by Act 26905 (n = 864).

Food Groups	Food Categories		Sodium Content (mg/100 mg)			Products on Target	
		Products (n)	Mean	Median	Maximum levels (Act 26,905)	n	%
**Meat and meat products**	Cooked sausages	69	914.5	950.0	1196	63	91.3
	Luncheon meat	32	942.2	918.8	1196	26	81.3
	Dry sausages	29	1496.8	1500.0	1900	24	82.8
	Fresh sausages	31	871.8	830.0	950	23	74.2
	Hamburgers	42	771.0	800.0	850	39	92.9
	Breaded chicken products	17	468.7	516.9	736	17	100.0
	**Total meat and meat products**	**220**	**927.4**	**844**	**--**	**192**	**87.3**
**Farinaceous**	Bran crackers	33	558.5	563.6	941	33	100.0
	Non-bran crackers	42	463.0	530.0	941	42	100.0
	Snack crackers	25	851.9	859.4	1460	25	100.0
	Corn flour snacks	12	685.7	700.0	950	11	91.7
	Cheese puffs	11	644.4	596.0	950	11	100.0
	Cheese-flavored sticks	7	745.7	752.0	950	7	100.0
	Potato chips	64	533.8	552.0	950	64	100.0
	Unsalted potato chips	1	10.4	--	950	1	100.0
	Salted peanuts	11	657.2	746.7	950	11	100.0
	Nachos	9	577.3	648.0	950	9	100.0
	Other snacks	24	649.5	688.0	950	23	95.8
	Dry sweet cookies	137	270.8	276.7	512	134	97.8
	Filled sweet cookies	98	195.5	203.3	429	97	99.0
	Wholemeal bread	47	421.0	400.0	530	41	87.2
	White bread	32	482.3	498.0	501	27	84.4
	Hot dog buns	10	461.1	467.0	501	10	100.0
	Hamburger buns	14	438.9	468.0	501	13	92.9
**Total farinaceous**		**577**	**423.1**	**400.0**	**--**	**559**	**96.9**
**Soups, bouillons, and dressings**	Bouillons	24	339.0	378.4	430	22	91.7
	Clear soup	11	310.2	302.8	346	10	90.9
	Cream soup	16	257.2	283.2	306	16	100.0
	Instant soup	16	230.7	228.3	352	16	100.0
**Total soups, bouillons, and dressings**		**67**	**288.9**	**294.0**	**--**	**64**	**95.5**
**All groups and categories**		**864**	**546.5**	**474.2**	**--**	**815**	**94.3**

**Table 3:** Comparison of sodium content in food groups and categories with the 2015 sodium reduction regional and lower targets (n 1375).

Food Group	Food Category	Products (n)	Regional Sodium Targets (mg/100 g)	Products above Regional Targets		Lower Sodium Targets (mg/100 g)	Products above Lower Targets	
				n	%		n	%
Soups	Wet and dry soups, as consumed	43	360	1	2.3	306	4	9.3
	Noodles in broth, as consumed	9	430	5	55.6	360	8	88.9
Meats	Meats and raw, cooked, and processed sausages	174	1210	12	6.9	690	140	80.5
	Dry cured meats and meats preserved at room temperature	40	1900	10	25.0	1350	30	75.0
	Breaded meat and poultry	17	735	0	0.0	470	9	52.9
Breads	Breads	110	600	7	6.4	400	40	36.4
Mayonnaise	Mayonnaise	35	1050	0	0.0	670	34	97.1
Cookies and biscuits	Cookies and sweet cookies	363	485	77	21.2	265	194	53.4
	Flavored cookies and crackers	25	1340	0	0.0	700	15	60.0
Cakes	Mixes for aereated cakes	11	400	6	54.5	205	10	90.9
Breakfast cereals	Breakfast cereals	85	630	6	7.1	500	14	16.5
Butter	Butter	19	800	1	5.3	500	1	5.3
Snacks	Snacks	139	900	4	2.9	530	96	69.1
Pastas	Pasta and noodles (dry, uncooked)	5	1921	0	0.0	1333	0	0.0
	Pasta and noodles, as consumed	259	640	34	13.1	440	71	27.4
Condiments	Meat and fish seasonings	19	23,000	1	5.3	21,775	1	5.3
	Bouillon cubes and powders *	22	20,500	11	50.0	18,000	15	68.2
**All groups and categories**		**1375**	**--**	**175**	**12.7**	**--**	**682**	**49.6**

* Bouillon cubes within the condiments group are added directly to food preparations and are not reconstituted with water.

## Supplementary Materials

The following are available online at https://www.mdpi.com/2072-6643/11/7/1474/s1, Table S1: Food product categories compared against National Act 26,905 maximum sodium levels (n 864).
