# Supplementary material for: Monitoring Sodium Content in Processed Foods in Argentina 2017–2018: Compliance with National Legislation and Regional Targets
Source: Nutrients. 2019 Jun 28;11(7):1474. doi: 10.3390/nu11071474 (PMC6682874; doi:10.3390/nu11071474)
Supplement: Supplementary file 1 [file nutrients-11-01474-s001.pdf]

Table S1. Food product categories compared against National Act 26905 maximum sodium levels (n 864).

Food groups	Food categories	Total products (n)	Products under maximum sodium levels		Products over target by % of excess							
					0-25%		25-50%		50-100%		>100%	
			n	%	n	%	n	%	n	%	n	%
**Meat and meat products**	Cooked sausages	69	63	91.3	2	2.9	3	4.3	1	1.4	0	0
	Luncheon meat	32	26	81.3	5	15.6	0	0	1	3.1	0	0
	Dry sausages	29	24	82.8	5	17.2	0	0	0	0	0	0
	Fresh sausages	31	23	74.2	7	22.6	0	0	0	0	1	3.2
	Hamburgers	42	39	92.9	3	7.1	0	0	0	0	0	0
	Breaded chicken products	17	17	100.0	0	0	0	0	0	0	0	0
**Total meat and meat products**		**220**	**192**	87.3								
**Farinaceous**	Bran crackers	33	33	100.0	0	0	0	0	0	0	0	0
	Non-bran crackers	42	42	100.0	0	0	0	0	0	0	0	0
	Snack crackers	25	25	100.0	0	0	0	0	0	0	0	0
	Corn flour snacks	12	11	91.7	1	8.3	0	0	0	0	0	0
	Cheese puffs	11	11	100.0	0	0	0	0	0	0	0	0
	Cheese-flavored sticks	7	7	100.0	0	0	0	0	0	0	0	0
	Potato chips	64	64	100.0	0	0	0	0	0	0	0	0
	Unsalted potato chips	1	1	100.0	0	0	0	0	0	0	0	0
	Salted peanuts	11	11	100.0	0	0	0	0	0	0	0	0
	Nachos	9	9	100.0	0	0	0	0	0	0	0	0
	Other snacks	24	23	95.8	0	0	1	4.2	0	0	0	0
	Dry sweet cookies	137	134	97.8	1	0.7	1	0.7	0	0	1	0.7
	Filled sweet cookies	98	97	99.0	0	0	0	0.0	1	1.0	0	0
	Whole meal bread	47	41	87.2	5	10.6	0	0	1	2.1	0	0

	White bread	32	27	84.4	4	12.5	0	0	0	0	1	3.1
	Hotdog buns	10	10	100.0	0	0	0	0	0	0	0	0
	Hamburger buns	14	13	92.9	0	0	1	7.1	0	0	0	0
**Total farinaceous**		**577**	**559**	96.9								
**Soups, bouillons and dressings**	Bouillons	24	22	91.7	2.0	8.3	0	0	0	0	0	0
	Clear soup	11	10	90.9	1	9.1	0	0	0	0	0	0
	Cream soup	16	16	100.0	0	0	0	0	0	0	0	0
	Instant soup	16	16	100.0	0	0	0	0	0	0	0	0
**Total soups, bouillons and dressings**		**67**	**64**	95.5								
**Total products**		**864**	**815**	94.3								
